# Cases Treated by Dr. Hesse, with Remarks by S. L.

**Published:** 1887-01

**Authors:** 


### CASES TREATED BY DR. HESSE, HAMBURG, WITH REMARKS BY S. L.

My good friend, Dr. Hesse, a disciple of the veteran Kunkel, publishes in the *A. H. Z.*, 17–19, some interesting cases, among others several of prosopalgia, with aggravations in the warm room, in bed, from hot food, disgust for fat, in the evening till midnight, from lying with the head low, or on the left side, or by lying on the side not affected; all such cases yielded promptly to Pulsatilla, and Hesse remarks that according to Bœnninghausen Pulsatilla is especially indicated by aggravation from *lying on the left side or on the side not affected.*

In Allen VIII, p. 239, we find under aggravations: After lying down, offensive odor from mouth; after lying down, cough, dyspnœa; in bed, twitching of muscles of thighs; tremulous sensation in legs and knees; after becoming warm in bed, pain in heels; after lying down, headache in side on which he does not lie; lying upon either side, spasmodic symptoms; lying upon left side, anxiety and palpitation; lying upon right side, cutting in lower rib. Lippe (*M. M.*, 562) gives us aggravation while lying with the head low, on the left, or on the painless side, and *amelioration while lying on the painful side,* whereas Pulsatilla shows, otherwise, amelioration in a cold place, in the open air, from eating cold things, from taking cold water in the mouth (toothache).

Teste (*M. M.*, 265) gives us, also, pains which manifest themselves principally on the side on which one is not lying, and quieted sometimes by lying on the affected side, alternating symptoms, aggravated by a sitting posture, by passing from a cool air into the warm air of the room.

Hahnemann (*M. M. Pura,* Dudgeon's edition, II, 349): Symptom 203: Toothache, commencing at two A. M., did not allow him to lay the head on a cold part of the bed (Hesse's case, 38, improved by pressing a cold iron to the face); 650, when she lies on left side she complains of anxiety and great palpitation of the heart and loses her breath; 895, the symptoms are ameliorated in the open air; 898, suffering from open air, he dreads it; 901, when lying on back the pains are diminished and go off, but when lying on either side they are aggravated or renewed. The editors remark that *the alternating actions a medicine most frequently displays, and which are most severe and most singular, are the most efficacious for the homœopathic cure of diseases.* Such an alternating action preponderates in Pulsatilla.

Piedvache (Jousset, *M. M.*, II, 524) says: Nocturnal symptoms prevail under Pulsatilla; patient feels better when lying on his back with his legs drawn up, and the pains increase or are renewed as soon as the patient turns to one or the other side. Motion, marching, change of position, gives ease, the contrary takes place only rarely. This author finds often great similarity between the symptoms of Pulsatilla and Ignatia, especially in neuralgic cases, as in both relief is found by change of position, but the chilliness, the nocturnal aggravation, a chlorotic state, plead in favor of Pulsatilla.

Bayes in his *Applied Homœopathy*, p. 142, says of Pulsatilla: Many curious, unexplained medical points does the action of this little plant confirm, and to some extent elucidate. Among these *is its especial action on the left side of the body*, hence, perhaps, its special fitness for the cure of female disorders, which chiefly elect for their site that side of their victims. Oversensitiveness, impressionability, together with venous congestions and their sequelæ, will be benefited by Pulsatilla.

May not this impressionability explain the instability of symptoms as found in Pulsatilla as well as in Ignatia, and though keynotes and modalities are valuable, it shows us again and again that only in the totality of the symptoms is our salvation, and that the mental symptoms often will turn the balance in favor of a remedy.

Case 39. A man of sixty-two years suffered five years ago from dropsy, which was cured by the late Dr. Goeze, and about two weeks ago his old trouble returned and rapidly increased, so that abdomen and legs are greatly enlarged. From some points of his legs water trickles down. Appetite good, no thirst, passes little urine; after eating pains in stomach. All of which was removed by Rhus tox.[3], thrice daily a drop. Though I found out afterward that he was sensitive to cold, damp weather, and preferred to lie propped up in bed; the selection of the remedy was based on a remark of Bœnninghausen in his *Aphorisms of Hippocrates:* Characteristic for Rhus: *Œdema of lower legs, with constant, profuse, involuntary trickling of water from ulcerating points, which will not form pus* (to be followed by Lycopodium).

How many, or rather how few, of us heard of this work of Bœnninghausen which contains lots of such good hints, for old Bœnninghausen was a close observer. The symptom as given by this author is not found in Allen's *Encyclopædia,* but the characteristic vesicular eruption with oozing of fluid is well known. Dunham in his lectures shows us that Rhus causes lassitude, languor, and weight, especially in lower extremities, prostration and emaciation, a serous infiltration of the cellular tissue in various parts, and an acrid state of the secretions generally. The individuality of the patient, sensitiveness to atmospheric influences, coincided so well with the individuality of the drug as given by Dunham, that Rhus was clearly indicated, though we also find under its symptoms purulent discharges. This individuality of the patient, we think, is too often neglected in the selection of the corresponding drug, and failures follow. Do we not witness the same reciprocity in proving a drug? A fails to witness any symptom from the drug; no matter how large or small the dose may be, the drug does not respond to his individuality; B gets only symptoms from doses of heavy calibre, whereas the impressible C shows us the finer action of the drug. Thankful, as we are, for what we receive in the *Cyclopædia of Drug Pathogenesy,* still we consider it fragmentary as long as the day-books of the latter are withheld, for only thus the totality of the remedy could be elucidated.

In cases 40–44, Hesse shows us the action of *Sepia.* This case, 40, reads: A tailor of thirty-seven years, with dark hair, pale, lean, complains of tearing in the teeth, in left shoulder, right leg; worse when sitting quiet or lying down; better during motion; feels unrefreshed in the morning, and dull of conception; he has to lie on his right side, as lying on the left side or on the back is unpleasant; eruption on legs, itching principally when he comes from the cold air into a warm room, and when sitting for a long time, as the legs then become restless, and he has continually to change position; appetite fair; constipation; feels too hot in-doors, and is inclined to melancholia. Cured by Sepia$^{30}$, a powder once a week.

Mrs. H., brunette. For some time pressure in the stomach, and drawing from the stomach through the right chest into the shoulder, setting in one hour after eating; relieved by loosening her dress, and eructations; appetite good; no thirst; hypochondriasis; falls asleep late; dreams constantly, and feels unrefreshed in the morning; tires easily, and feels worse in snowy weather. Sepia [30] cured.

A man about thirty complains for the last four weeks of pains in the small of the back after exposure to wet and cold weather; worse in the morning, when sitting, and when beginning to move about; falls asleep late, and cannot lie on left side or on his back; vertigo in the morning when rising, and feels uncomfortable in a hot room or when sitting too long; perspires easily. Sepia [30], a powder every morning and evening, cured.

We differ from Dr. Hesse when he says the differentiation between Rhus and Sepia in such cases is easy. If we take Gross' *Comparative Materia Medica* for our only guide, there is too much of "better or worse" in either drug, but when we study the individuality of each drug the differentiation lies open before our eyes. Father Hahnemann puts Rhus in the *Materia Medica Pura*, and Sepia in his *Chronic Diseases;* hence an antipsoricum, with its vitality below par, sluggishness of the circulation the keynote to its application, and the effects of a single dose of Sepia often lasts for many weeks. If Rhus is often the indicated remedy in rheumatic affections, we find Sepia more often needful in rheumathritis and gout, to which many a time we find added an atonic dyspepsia, with its plethora of symptoms, so well covered by Sepia. Though we meet in both aggravation from repose and amelioration from motion, how different is its application; in Rhus we read: greatest rigidity and pain on first moving the joints after rest and on waking up in the morning; relieved by moderate exercise; the cold, fresh air is not tolerated; sleep is restless from tumultuous coursing of the blood, whereas the Sepia patient hates the warm room, dislikes the heat of the bed, and feels best in the fresh air; his sleep is therefore wakeful and unrefreshing. Get that liver right with your Sepia, get assimilation in order by Sepia, consider female affections as constitutional disorder and rectify them by Sepia, if indicated according to the totality, and Homœopathy will report many a victory; but allow this precious drug time to act and do not interfere.

Case 44 is one of palpitation of the heart cured by Sepia, and Case 45 another one cured by Magnesia muriatica. Let us compare them:

A man about thirty, stout and healthy looking, complains of palpitations and constipation; appetite good; no thirst; after eating bloatedness of abdomen, anguish and palpitation, especially when no alvine discharge took place for some time. He complains of the same troubles when sitting down after having had a walk, never during walking. Flatulency on the left side of the abdomen rising upward, relieved by pressure. Hates to be sitting for some time, as it aggravates the palpitations, and still enjoys dancing the night through without suffering. Worse in cold wind; cannot lie on left side, prefers to walk with head uncovered; sweats easily, especially on the feet, which often feel cold. After the failure of Lycopodium, Sepia cured.

An elderly brunette lady complains of palpitation when quiet or when lying on her right side. Exercise, especially in the fresh air, feels good. Bowels move daily, but with difficulty and unsatisfactorily. Magnesia muriatica$^{30}$, a dose off and on, cured her entirely, though the remedy sometimes produced constipation.

Thanks to Schüssler, the Magnesia and Sodium salts are not as much neglected any more as they were of yore, and if all of them are not yet installed into the regular army of proved remedies, let us not forsake Hering's breech presentations and accept clinical facts at their value, if we even must allow some discount (Magnesia phosphorica). Hahnemann, in his *Chronic Diseases*, says: Experience has taught me to look upon Magnesia mur. as a most valuable antipsoric. I recommend it, among others, for aching pain in the liver, even when walking or when lying upon the right side; knotty, hard, insufficient, delaying stool; palpitation of the heart when sitting and when rising from a seat, going off during motion; oppression of the heart; sleep does not refresh, feels weary in the morning; great restlessness and tossing about at night; she feels too hot to fall asleep. Mr. Clifton's remarks (Hughes' *Pharmacodynamics*) are very appropriate, for he found the drug very beneficial in congestion and enlargement of the liver. They all occurred in women whose uterine health was imperfect, and had been connected for months or years with recurring attacks of indigestion, biliousness, constipation, with large round motions, like balls, and inability to lie on the right side. Dr. Guernsey considers it one of the most important remedies in hysterical conditions. (Dr. Deschere lately cured a most aggravated case of gastralgia, which had lasted for months and obstinately refused to yield to narcotics and the washing out process, with one dose of Magnesia mur.$^{200}$) Uterine spasms followed by leucorrhœa are often speedily cured by this medicine.

Sepia and Magnesia muriatica are so nearly alike that it may often be difficult to differentiate between them. We may, perhaps, take here a hint from Schüssler, who finds the Magnesium phosphate so valuable as a nerve remedy. We find far less of this action on the nervous system in Sepia, where venous congestion and an atonic, torpid condition of the female sexual organs prevail. Mere symptom covering will not do; we learn more and more that we have to study out the central point, the sun, as it were, of a drug, and the other symptoms will then rally around it, like satellites around their chief. Only thus can the constant cry of an ever-increasing materia medica be answered and the study and application of our materia medica be based on a surer foundation. We are not too old to learn a better way, if there is any, and should we be wrong, we would be only too glad to be led on the road where mistakes in prescribing are the exceptions.

